# Uncovering the molecular and physiological processes of anticancer leads binding human serum albumin: A physical insight into drug efficacy

**DOI:** 10.1371/journal.pone.0176208

**Published:** 2017-04-20

**Authors:** Chuanbo Liu, Zuojia Liu, Jin Wang

**Affiliations:** 1 State Key Laboratory of Electroanalytical Chemistry, Changchun Institute of Applied Chemistry, Chinese Academy of Sciences, Changchun, Jilin, P.R. China; 2 University of Chinese Academy of Sciences, Beijing, P.R. China; 3 Department of Chemistry and Physics, State University of New York, Stony Brook, New York, United States of America; Islamic Azad University Mashhad Branch, ISLAMIC REPUBLIC OF IRAN

## Abstract

Human serum albumin (HSA) has its ability to bind drug molecules and influence their efficacies. Although anticancer leads NSC48693 and NSC290956 functioned at the same mechanism, the drug efficacies were obviously distinct. To gain insight into the distinct drug efficacy, the molecular and physiological processes of anticancer leads binding HSA have been investigated via a combined experimental and theoretical approach. The binding site, as characterized by fluorescence quenching and molecular modeling, is found to be located at site II in subdomain III A for NSC48693 with tight binding and at site FA1 in subdomain I B for NSC290956 with negatively cooperative binding, respectively. As indicated by the thermodynamic analysis, NSC48693 binds to HSA with an enthalpy driven mechanism, while NSC290956 binding with HSA is entropically driven. The further kinetic analysis indicates that the association rates appear to be similar to these two anticancer leads, however, the dissociation rate of NSC48693 is approximately 5-fold slower than that of NSC290956. For NSC48693, the pharmacodynamic efficacy is less than that of NSC290956, while its pharmacokinetic behavior is better than that of NSC290956. These parameters influence the pharmacodynamic efficacy and pharmacokinetic behavior, which will give further impacts on drug efficacy *in vivo*.

## Introduction

The high effective binding affinity and specificity between chemotherapy agents and biomacromolecules are increasingly explored in pharmaceutical research field. Recently, we have initiated an *in silico*-based specificity-affinity virtual drug screening [1,2] and successfully picked out anticancer leads NSC48693 and NSC290956 (S1 Fig) that displayed promissing bioactivity against pancreatic cancer cell lines [3,4]. Although they functioned at the same potential mechanism, the pharmacodynamic efficacy is obviously distinct. It is well known that *in vivo* drug efficacy is determined not only by pharmacokinetics but also by pharmacodynamics, which can be influenced by absorption, distribution, metabolism and excretion. These processes are greatly related to the drug-plasma protein binding property [5]. In most cases, plasma protein binding strongly affects drug distribution, elimination rate and pharmacokinetic behavior, which ultimately leads to distinctly clinical outcome. In principle, only the free (unbound) drugs can permeate biological membranes and exert their bioactivities. Conversely, drugs bounnd to plasma protein reduce the permeability and therefore enhance retention effect. This gives the opportunity to selectively accmulate drugs in disease tissues, resulting in great increase in the local drug concentration and reduction of the toxity to the normal tissues [6]. In view of this, the molecular and physiological processes of anticancer leads binding plasma protein should be best elucidated experimentally to gain basic molecular information for offering physical insight into increased mechanistic understanding of the pharmacodynamics and pharmacokinetics.

Up to date, there has been extensive investigations on the interactions of various ligands with plasma proteins [7] and the impacts on *in vivo* drug efficacy have also been discussed [8, 9]. It is generally accepted that the protein-drug interactions should be examined when the bound fraction exceeds about 95% of the total drug concentration which tends to have low therapeutic index [10]. HSA as the most prominent protein in plasma [11] and the best known receptor protein for its extraordinary ligand binding capacity [12] attracts the most attention [13–16]. The remarkable carrier function makes drug-HSA interaction a major factor involving in both pharmacodynamic efficacy and pharmacokinetic behavior. The applications of high-throughput assay and X-ray crystallographic method have clearly clarified the distinct binding sites and the principal ligand binding regions [17]. We have reported a combined computational and experimental approach to identify the underlying mechanism (i) HSA does possess the carrier function and (ii) site I serves as a primary binding site for most small molecules [18]. As far as small molecules with different chemical structure are concerned, however, the prediction of ligands-HSA binding mechanism is still challenged by binding promiscuity due to the inherent flexibility of HSA and the ligand-binding cooperativity. Besides, the plasma protein-protein interactions under the influence of external chemical compounds are also being covered [19, 20]. The dynamic structure that are emerged by the interactions of plasma proteins exhibited even more complexity.

The main focus of this study is to examine the occurrence and nature of both NSC48693 and NSC290956 binding HSA via a combined experimental and computational approach for uncovering their distinct molecular and physiological processes. Experimentally, ultraviolet visible absorption spectroscopy, steady-state fluorescence quenching, and isothermal titration calorimetry measurements were performed to characterize the binding mechanism. Far-ultraviolet visible circular dichroism spectroscopy was collectively employed to confirm HSA conformation change upon ligand binding. Moreover, surface plasmon resonance assay provided pharmacokinetics information on the two anticancer leads binding to HSA. Computational modeling revealed that the binding sites were distinct due to the different chemical properties. The current research not only advances the understanding to this unique carrier protein HSA but also ultimately offers a new physical insight into the advancement of pharmacodynamics and pharmacokinetics prediction-method.

## Materials and methods

### Chemicals

Fatty acid free HSA lyophilized powder (Cat#A3782) was purchased from Sigma-Aldrich (St. Louis, MO). NSC48693 and NSC290956 were kindly supplied from NCI/DTP Open Chemical Repository (http://dtp.cancer.gov) and confirmed by HPLC-MS before further usage. Hydrophobic NSC290956 was resolved in DMSO as stock solution and 25 mM MES reaction buffer (pH 6.0) was used to increase its solubility. Hydrophilic NSC48693 was resolved in 0.01 M PBS (pH 7.4) throughout the experiment. HSA stock solution was prepared in either MES or PBS buffer in the presence of DMSO (v/v ≤ 1%). HSA concentration was spectrophotomerically verified in a UV-5800 Spectrophotometer (METASH, China). All chemicals were analytical purity and double distilled water was used throughout.

### Ultraviolet-vis spectroscopy measurement

Ultraviolet visible (UV-vis) absorption spectra of either 10 μM small molecule alone or the mixture of HSA-anticancer leads were recorded on a double beam Cary 500 scan UV spectrophotomer (Varian, Japan) at 298 K in the range of 200–500 nm using a quartz cell with 1.0 cm pathlength. Baseline was corrected before each scanning.

### Fluorescence quenching measurement

Steady-state fluorescence spectra were recorded on an Eclipse fluorescence spectrophotometer (Agilent Cary, USA) with a 1 cm path length quartz cuvette at 293 K and 298 K, respectively. Sample temperature was kept within ± 0.2 degree by recycled water throughout the experiment. The excitation and emission band passes were all set at 5 nm by keeping the excitation wavelength at 290 nm to achieve maximum fluorescence emission. The emission spectra was measured in the region of 305–450 nm, and the fluorescence intensity at peak position 340 nm was extracted for further analysis. HSA was diluted from stock solution with reaction buffer to a concentration of 10 μM for each quenching experiment. Titration was performed by successive addition of 1 mM anticancer leads into quartz cuvette, and the minor intensity decrease due to volume change was corrected by the control experiment.

In Fluorescence quenching, the inner filter effect (IFE) is of importance due to its effect on the emission intensity [21]. One of the biggest causes of the IFE is the re-absorption of the emitted light in the excitation and emission region. It is an important issue that these effects cause severe underestimation of the fluorescence intensity measured because the concentration of the quencher increases when a Stern-Volmer analysis is carried out, and therefore the ability of the quencher to absorb the emitted light will also increase. In this case, since HSA does not have any absorption beyond 305 nm, the correction factor is used to correct these artificial effects [22]:

$$F_{c}=F_{0}\left\{\frac{anti\text{log}\left(A_{em}+A_{ex}\right)}{2}\right\}$$

Where *A*_*em*_ and *A*_*ex*_ represent the absorbance of small moecule at emission and excitation wavelength, respectively. *F*_0_ and *F*_*c*_ represent the observed fluorescence intensity and corrected fluorescence intensity, respectively.

### Synchronous fluorescence measurement

Synchronous fluorescence spectra were recorded on a FluoroMax-4 spectrophotometer (HORIBA, Japan) by keeping the temperature at 293 K. Sample preparations and experiment conditions were the same as described above in the fluorescence quenching measurement. Spectra were obtained by simultaneously scanning excitation and emission spectra wavelength from 200–600 nm with step increment of 1.0 nm by fixing the wavelength interval between emission and excitation at 15 nm and 60 nm, respectively.

### Circular dichroism spectroscopy measurement

Circular dichroism (CD) spectra were measured with a J-820 spectropolarimeter (JASCO, Japan) under constant nitrogen flow rate. 2 mm light path length quartz cuvette was employed for far-UV (200–250 nm) measurement in the presence of HSA at 2 μM. Each spectrum was an accumulation of three succession scans at the conditions of 0.1 nm wavelength resolution, 200 nm per minute scan speed and 1.0 nm bandwidth. All measurements were conducted at room temperature.

#### Binding thermodynamic measurement

The calorimetry measurement was carried out by isothermal titration calorimetry (ITC) (TA Instruments, USA) at 310 K. HSA in different reaction buffers (0.01 M PBS and 0.025 M MES) were extensively dialyzed before titration. All solutions were degassed afterward and the sample cell was filled with 97 μM of 300 μL HSA solution for NSC48693 and 75 μM of 300 μL HSA solution for NSC290956, respectively. Anticancer leads were dropwise injected into the cell with a volume of 2.5 μL at concentration 1 mM for NSC48693 and 600 μM for NSC290956, respectively. The titration was afterward started with continuously stirring at 250 rpm. The raw heat rate data were integrated under injection peaks to obtain the reaction heat of each injection after substracting dilution heat which was measured by injecting with a blank control titration. The calculated heat changes were analyzed using the NANOanalyze software (TA Instruments, USA) and replotted with Origin 8.5. The free energy change (Δ*G*), enthalpy change (Δ*H*) and entropy change (Δ*S*) were calculated according to the thermodynamic formulas below:

$$\Delta G=-RT\text{ln}K_{A}=\Delta H-T\Delta S$$

Where *K*_*A*_ is the association constant at the absolute temperature *T* (310 K) and *R* represents the gas constant (8.3151 J mol^-1^K^-1^).

#### Binding kinetic measurement

Binding kinetic measurement was performed using Reichert 4-chanel SPR instrument Reichert4SPR (Reichert, USA). HSA was immobilized on carboxymethyl dextran chip at the condition of 0.1 mg/mL protein in 10 mM sodium acetate (pH 4.5). The final density is about 12,000 RU and flow rate is 20 μl/min. During immobilizing, the sensor chip was preconditioned with 3-min injection of double diluted water. Thereafter, the first flow cell surface was activated through using 0.05 M N-hydroxysuccinimide (NHS)/0.2 M 1-ethyl-3-(3-dimethylaminopropyl)carbodiimide hydrochloride (EDC) for 7 min, and then functionalized by injecting a solution of HSA (0.1 mg/mL) in 10 mM sodium acetate buffer (pH 4.5) for 20 min. Finally, unreacted NHS groups were deactivated with 1 M ethanolamine (pH 8.5) for 10 min. Simultaneously, the control flow cell was treated using the same conditions as described above. For binding analysis, a 2-fold dilution for each concentration gradient series of NSC48693 (100~6.25 μM) was injected for 60 s binding and 100 s dissociation. For NSC290956 (50~3.125 μM), experiment was treated with 90 s binding and 90 s dissociation time interval. Final data obtained were corrected for instrumental and bulk artifacts by double referencing to a control sensor chip surface and blank buffer injection. The binding responses were concentration-dependent and dissociation constant *K*_*D*_ values were derived from 1:1 kinetic model as well as steady state analysis using TraceDrawer software (Reichert, USA).

#### Molecular modeling

Molecular docking were carried out on the ligand-HSA system using Autodock4 [23], which possesses a free-energy scoring function based on a linear regression analysis. The AMBER force field and a larger set of diverse protein-ligand complexes with known inhibition constants were used in the simulation. The standard error is around 2.5 kcal/mol, which enough discriminates leads with milli-, micro- and nano-molar inhibition constants. The crystal structure of HSA used for molecular docking was retrieved from Protein Data Bank (PDB ID: 1H9Z). And 3D structures of two leads were built by using the web-tools corina3D. Water molecules were removed from the structure and the missing atoms of side chain were added using Swiss PDB Viewer. Autodock tools were used to prepare protein and ligands. HSA and ligands were protonated and Gasteiger partial charges were assigned. A set of grids of 40×40×40 Å with 0.375 Å spacing was calculated around each docking site using AutoGrid. The non-polar hydrogen atoms were merged for the protein and ligands. The part of myristic acid retained as a “plug” in the original position was prepared by using the Chimera package [24], adding the hydrogen atoms and the AM1BCC charges [25]. The docking results from each of the 500 calculations were clustered and ranked on the basis of root-mean square deviation and the binding free energy, respectively.

## Results

### 1. Characterization of the interactions between anticancer leads and HSA

**Fig 1:**
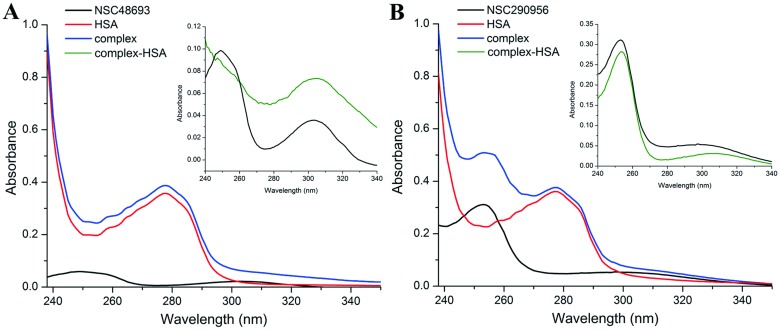
UV-vis absorption spectra of HSA in the presence of NSC48693 (A) and NSC290956 (B). The inserted figures represent the difference spectra i.e. complex spectrum minus small molecule spectrum. The colored lines represent the absorption spectra of individual component as indicated.

UV-vis absorption is a powerful method to explore the complex formation. On the one hand, the absorption spectra measured is directly related to the molecular energy levels that are being examined. On the other hand, peaks and valleys can be viewed as the ‘finger print’ of the analytes. The formation of binding complex therefore can be characterized by the changes in UV-vis absorption spectra. NSC48693 alone has two maximum absorption peaks located at 249 nm and 304 nm as displayed in the figure inserted in Fig 1A. By contrast, in the NSC48693-HSA complex, the 249 nm peak radically disapear and the 304 nm peak shows an appreciable enhancement of absorption intensity concomitant with a slightly bathochromic shift to 305 nm (Fig 1A). For the NSC290956-HSA complex system, there is a full spectrum decrease of absorbance as shown in the picture inserted in Fig 1B. Moreover, the major peak located at 253 nm is bathochromic shift to 254 nm (Fig 1B), indicating certain interactions occur between NSC290956 and HSA. In both cases, the UV absorption intensity of HSA at 278 nm is significantly enhanced with the addition of anticancer leads. In theory, the red-shift of the absorption peak most likely emerges when *π* electron accumulation between the embedded molecules and the protein base occurs, thus resulting in lower π→π* transition energy [26]. Accordingly, the spectrum shape and absorption intensity change of HSA in anticancer leads-HSA complex systems can be interpreted as the result that NSC48693 and NSC290956 binding to HSA alters the microenvironment around HSA [27]. Collectively, the data strongly suggest that the interactions between anticancer leads and HSA occur and anticancer leads accommodate to respective binding site on HSA with various binding behavior.

**Fig 2:**
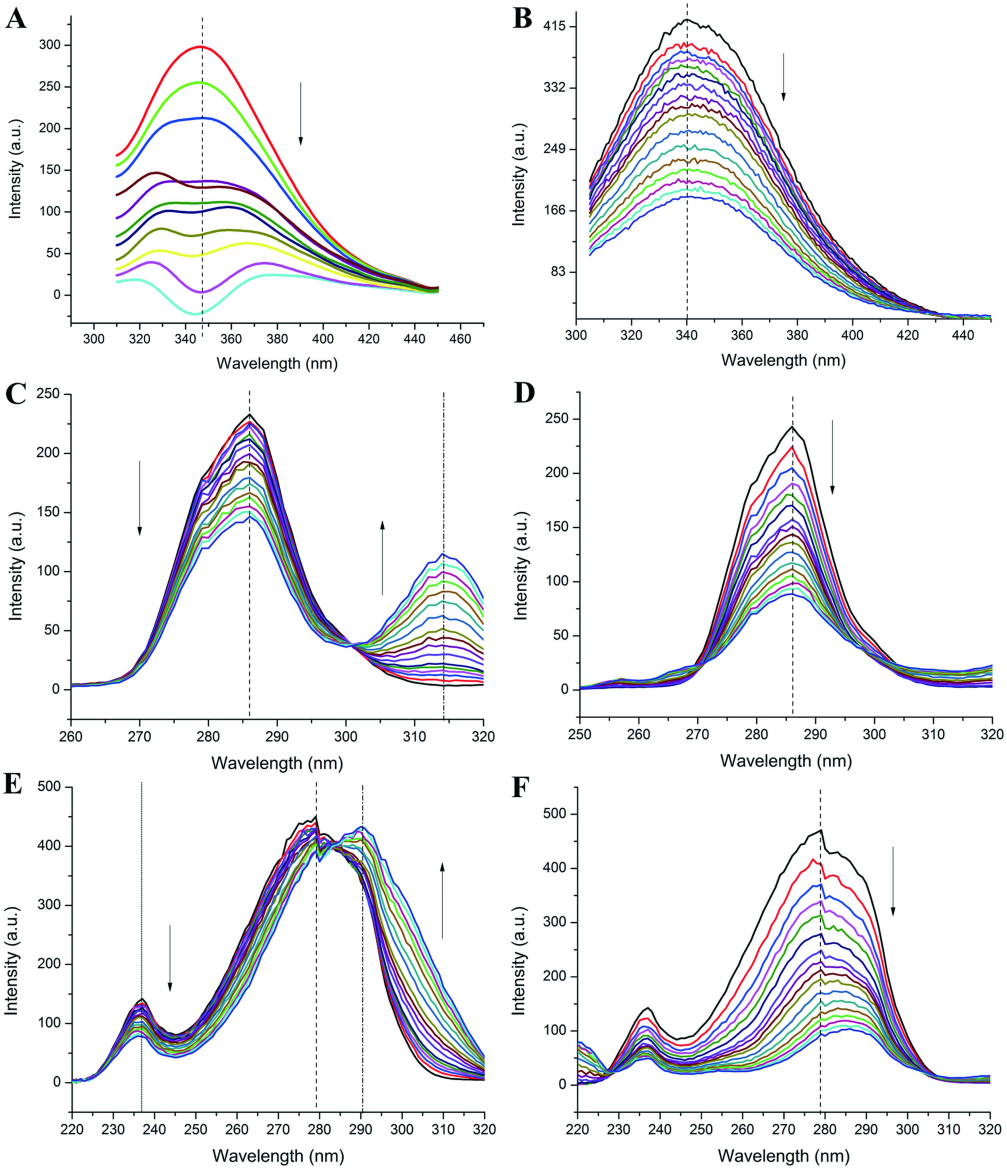
Steady-state fluorescence spectra of HSA in the presence of NSC48693 (left lane) and NSC290956 (right lane). Fluorescence quenching spectra of HSA in the presence of NSC48693 (A) and NSC290956 (B). A represents a fluorescence quenching difference spectra: HSA-NSC48693 complex spectra subtracting alone NSC48693 spectra. Synchronous fluorescence quenching spectra of HSA in the presence of NSC48693 (C) and NSC290956 (D) at Δλ = 15 nm. Synchronous fluorescence quenching spectra of HSA in the presence of NSC48693 (E) and NSC290956 (F) at Δλ = 60 nm. Calculated peak positions are shown with dashed lines, and labeled arrows indicate the concentration increasing of anticancer leads.

The aromatic amino acid fluorescence quenching has been used as an effective method to deduce binding affinity and binding mechanism among ligand-protein interaction [27]. HSA shows a characteristic emission maximum at 340 nm when it is excited at 290 nm wavelength, and the sole tryptophan (W214) residue located at site I in subdomain II A is the reason for this intrinsic fluorescence phenomenon. Since tryptophan is a polor fluorophore, its intrinsic fluorescence is very sensitive to microenvironmental hydrophobicity. NSC48693 emits photons when exicted at 295 nm (S2 Fig). The fluoresence intensity of NSC48693 is substracted from the fluorescence intensity of the NSC48693-HSA system to obtain the fluorescence quenching difference spectrum (FQDS). This reflects the extent of the fluorescence quenching when NSC48693 was titrated into HSA solution. As shown in the FQDS (Fig 2A) and fluoresence quenching spectrum (Fig 2B), the fluorescence intensity of HSA decreases with progressively titrating NSC48693 and NSC290956 into HSA solution. It suggests that the emission light of HSA is quenched by both NSC48693 and NSC290956. Simultaneously, the binding of NSC290956 with HSA produces a red-shift effect, moving the maximum peak position from 340 nm to 345 nm. However, little noticeable peak movement is observed in the FQDS of NSC48693. Since the emission fluorescence is strongly dependent on the local microenvironment, the stronger the polarity, the larger the solvent relaxation effect. This principle ultimately results in a longer emission wavelength, which is therefore used to estimate the surrounding hydrophobicity of the polar fluorophore. Obviously, the binding interaction of NSC290956 with HSA alters the microenvironment of W214. This leads to an observable change of the intrinsic fluorescence spectra, which is benificial to analyze the binding event [28]. The red-shift of maximum emission wavelength indicates that W214 is exposed to water environment due to HSA bound NSC290956. Under the experimental temperatures and pH conditions, HSA conformation is not affected [29], and so the structure change is out of the concern. We can therefore safely reach a conclusion that the pocket of site I adjacent to W214 is slightly opened towards a more hydrophilic environment as a result of NSC290956-HSA interaction. However, wavelength movement is not observed in the NSC48693-HSA system, suggesting the binding does not change the local environment of site I [29].

The synchronous fluorescence spectra can provide valuable information of the molecular environment around in a vicinity of the chromosphere molecules through measuring the fluorescence quenching and the maximum emission wavelength shift. In this study, the excitation-emission wavelength difference Δλ was stabilized at either 15 nm or 60 nm, representing the characteristic of tyrosine or tryptophan residue, respectively [30]. Although NSC48693 can be excited by 295 nm incidence light with a maximum emission wavelength of 350 nm (S3A Fig), the addition of NSC48693 keeps the emission maximum of tyrosine at the same position by fixing Δλ at 15 nm. In contrast, the fluorescence intensity of HSA is decreased dramaticly (Fig 2C), while NSC48693 titrated into blank buffer does not produce any difference curve (S3B Fig). These results strongly indicate that the quenching of fluorescence intensity can be completely attributed to tryptophan quenching of HSA, as shown in the synchronous fluorescence spectra by fixing Δλ at 60 nm (Fig 2E). However, the interaction of NSC48693 with HSA fails to altere the local microenvironment of the sole tryptophan residue (W214). Thus, the combined data suggest the binding site is not located at site I that has the sole W214 residue of HSA. Conversely, NSC290956 binds to HSA with a distinct manner as shown in Fig 2D and 2F. The emission intensities of both tyrosine and tryptophan residues are quenched with increasing concentration of NSC290956. It is clear that a large tryptophan emission wavelength shift from 279 nm to 286 nm is observed by fixing Δλ at 60 nm, whereas the wavelength of tyrosine keeps at the position by fixing Δλ at 15 nm. Considering the steady-state fluorescence spectrum, the data here indicat that binding of NSC290956 with HSA alters the local sturcture of site I (subdomain II A), but the binding site is not at site I. As a final note of caution, however, it is worth mentioning that there are probably other binding modes but most of them are so unstable that they disappear quickly and the fluorescence quenching of HSA carries through the dynamic equilibrium.

### 2. Binding mechanism and binding constants

**Fig 3:**
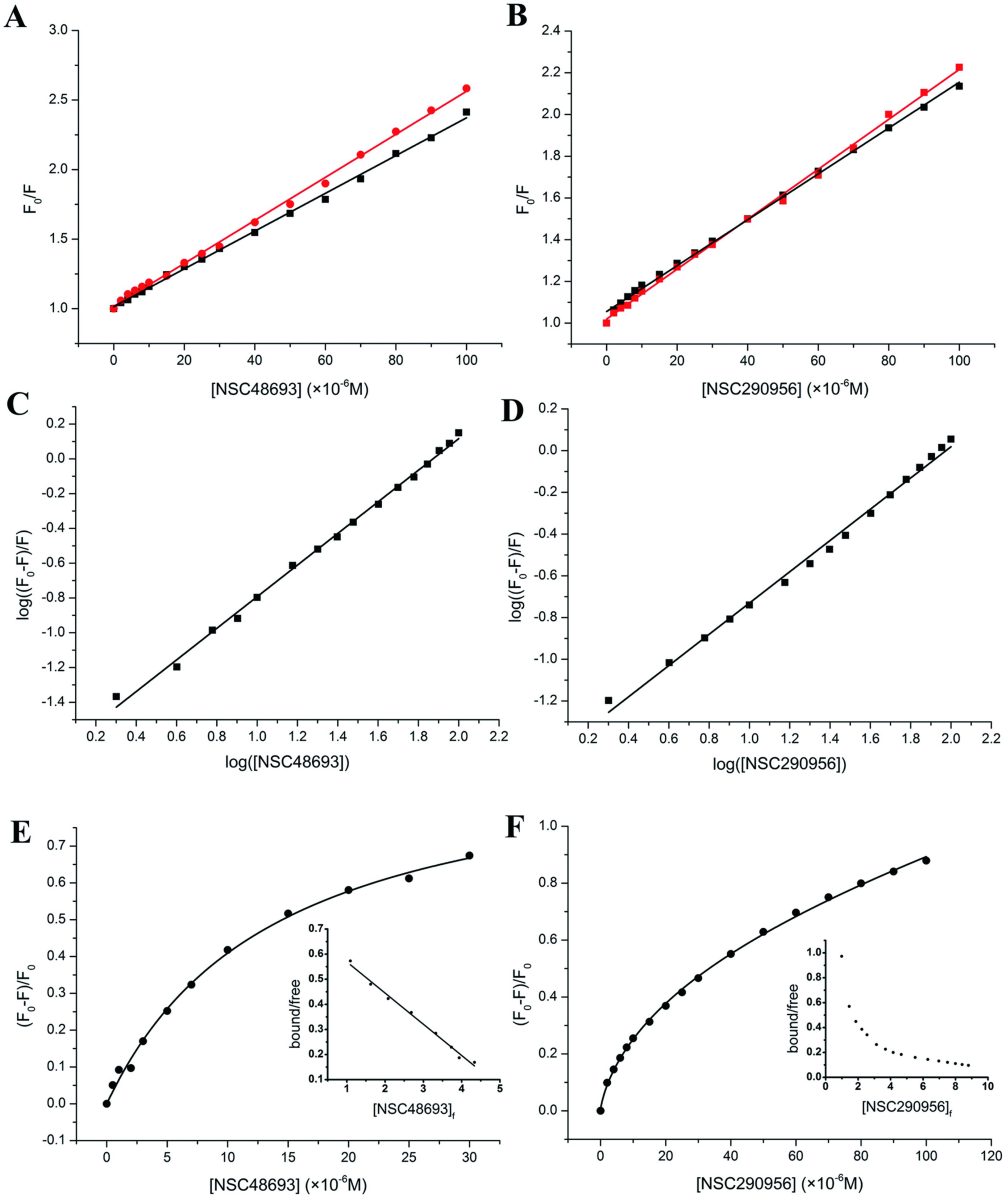
Binding constants and binding mechanisms. Stern-Volmer curves for quenching various concentrations of NSC48693 (A) and NSC290956 (B) with HSA at 293 K (black dots) and 298 K (red dots). Modified Stern-Volmer curves for quenching various concentrations of NSC48693 (C) and NSC290956 (D) with HSA at 293 K. Nonlinear regression fitting curves for quenching various concentrations of NSC48693 (E) and NSC290956 (F) with HSA at 293 K. The inserted pictures show the Scatchard plots of each binding data.

In an attempt to gain insight into the molecular mechanism of HSA association, we have determined the binding mechanism, binding constant, binding site, and the number of binding sites. As shown in Fig 3A, the plots of fluorescence quenching show linear relation at each experimental temperature, indicating that NSC48693 can be well fitted to linear Stern-Volmer relationship:

$$F_{0}/F=1+Kq\;\tau_{0}\left[Q\right]$$ (1)

Where *F* and *F*_*0*_ is the steady state fluorescence intensity in the presence and absence of quencher, respectively; *Kq*, τ_0_ and [*Q*] is the quenching rate constant, the average lifetime of the molecule without quencher and the concentration of quencher, respectively.

By linear fitting, the quenching rate constant *Kq* can be obtained by the slope of Stern-Volmer curves. The *Kq* value is with small deviation at low ligand concentration, which could be attributed to the depletion effect of low ligand concentration compared with protein concentration [31]. These data indicate that only one type of quenching mechanisms is predominant for NSC48693, either dynamic one or static one [18]. Taking fluorescence lifetime of tryptophan in HSA at around 10^−8^ s [18], the corresponding constant of *Kq* is found to be 1.36 ± 0.016 × 10^12^ M^-1^s^-1^ for NSC48693. The maximum scattering collision quenching constant *Kq* of various quenchers for biopolymers is around 2.0 × 10^10^ M^−1^ s^−1^.^10^ In this study, *Kq* is much higher than the maximum value of collision quenching *Kq*. This means that the quenching is not initiated by dynamic collision but by static one, i.e. the association of NSC48693-HSA. In Fig 3B, the Stern-Volmer plots of NSC290956 show slightly downward curve towards X-axis at 293 K but convert to linear plots at 298 K. This result suggests that the mechanism of NSC290956 binding HSA is transformed from a cooperatively binding to a non-cooperatively binding with increasing temperature. The *Kq* for NSC290956 is found to be 1.10 ± 0.014 × 10^12^ M^-1^s^-1^, suggesting that the quenching is initiated by the association of NSC290956 to HSA. In both cases, the slope representing the fluorescence quenching rate is increased with increasing temperature. As far as the static quenching is concerned, however, the increasing temperature will weaken the ground state association and break the association bonds, which finally leads to less fluorescence quenching [18]. The seemingly paradox phenomenon can be explained by the following rationals: (i) the temperature increasing is not enough to break the covalent or non-covalent chemical bound; (ii) with the temperature increasing the diffusion coefficient is also increased, which therefore facilitates the formation of the encontered complexes between anticancer leads and HSA.

Modified Stern-Volmer plots are shown in Fig 3C and 3D, showing good linearity to modified Stern-Volmer equation:

$$\text{log}\left[\left(F_{0}-F\right)/F\right]=\text{log}K_{A}+n\text{log}\left[Q\right]$$ (2)

The intercept of modified Stern-Volmer plots is a quantification of the accessibility of the quencher, representing the extrapolation to infinite quencher concentration. The binding constant *K*_*A*_, 3.31 ± 0.16 × 10^4^ M^-1^ for NSC48693 and 1.99 ± 0.17 × 10^4^ M^-1^ for NSC290956, can be obtained by fitting quenching data to Eq (2). The linearity of both curves indicates that both NSC48693 and NSC290956 bind independently to one class of binding sites on HSA. The values of *n* are noticed to be 0.91 ± 0.013 and 0.76 ± 0.05 for NSC48693 and NSC290956 at 293 K, respectively. n is the reaction stoichiometric number for each small molecule, reflecting the number of accessible binding sites on protein surface. If n > 1, more than one binding sites are available. While if n < 1, the fluorescence quenching sites are only partially filled through binding to small molecules. From the data of *n*, it can be concluded that there is one independent binding site on HSA for NSC48693, but only 75% of HSA fluorescence are accessible to quencher site for NSC290956. Unexpectedly, quenching data is not saturated by using high concentration of NSC290956. This suggests that binding of NSC290956 to HSA is composed with non-specific binding.

The linear method provides considerable information about the binding events such as the binding affinity, the number of binding sites, and the enlightened quenching mechanism. The method involves in the linear transformation of binding equation to achieve a linear relationship. The linear transformation lacks the ability to analyze binding isotherms, which yet inevitably introduces statistical errors due to weighting effects. Given this fact, nonlinear regression fitting is also performed via an empirical formula (3) [32], which minimizes the distortion of inherent error to the linear form by considering nonspecific binding, multiple-binding sites and ligand cooperativity.

$$\frac{F_{0}-F}{F_{0}}=\frac{B_{\text{max}}{\left(K_{A}[Q]\right)}^{h}}{1+{\left(K_{A}[Q]\right)}^{h}}\text{+}P_{nsb}\times [Q]$$ (3)

Where *F* and *F*_*0*_ are the steady state fluorescence intensity in the presence and absence of quencher, respectively; [*Q*] is the concentration of quencher, *B*_max_ is the maximum independent binding site classes, *K*_*A*_ is the effective binding affinity; *h* is the Hill coefficient that describes the degree of ligand binding cooperativity and *P*_nsb_ is the nonspecific binding coefficient.

The nonlinear regression fitting was performed at the condition of only one class of binding sites presented in the system. As shown in Fig 3E, it can be clearly seen that the scatchard plots of NSC48693 are well fitted to a straight line, suggesting that this small molecule binds HSA in a non-cooperative behavior. More importantly, the Hill coefficient *h* is close to 1.0, indicating NSC48693 binding HSA is a single independent site. On the contrary, although the scatchard plots of NSC290956 are unable to be linearly fitted, these data can be fitted to the Hill equation (Fig 3F). The Hill coefficient for NSC290956, *h* = 0.7, is in good accordance with the result obtained by the modified Stern-Volmer plots. Aside from sample heterogeneities, the possible interpretations for nonlinear scatchard plots involve (i) multi-site models, (ii) negatively cooperative models, or (iii) multivalent ligand models. It is noted that these models are not mutually exclusive. Since the chemical structure of NSC290956 is simple (S1 Fig), multivalent ligand model is first ruled out from this case. Moreover, nonlinear regression fitting is significantly better than a two-site model. For two sites model, the coefficient of determination is 0.9551, but this value is 0.9991 for one site negatively cooperative model. In conclusion, NSC48693 recognizes HSA via binding to site II in subdomain III A on HSA with a single independent site, while NSC290956 recognizes HSA to an adjacent position at site I in a negatively cooperative manner. These results demonstrate a striking correlation between affinity and binding site, which largely depends on chemical structure of ligands.

### 3. Interaction forces between anticancer leads and HSA

Since the dependence of binding constant on temperature is revealed by steady-state fluorescence quenching, the thermodynamic parameters are further analyzed to uncover the type of interaction forces of anticancer leads-HSA complexes. Usually, the types of interaction forces between small molecules and macromolecules mainly include hydrogen bonds, van der Waals forces, electrostatic interactions and hydrophobic interactions [18]. To estimate the binding mode, the thermodynamic parameters, Δ*H*, Δ*S* and Δ*G* are often considered. On the basis of the characteristic signs of the thermodynamic parameters, the interaction forces between small molecule and protein can be assigned to hydrophobic interaction (Δ*H > 0*, Δ*S > 0*), van der Waals force (Δ*H < 0*, Δ*S < 0*) and electrostatic attraction (Δ*H < 0*, Δ*S > 0*) [18]. The entropy and enthalpy can be considered constant at a constant temperture, which is regulated by the van’t Hoff equation:

$$\text{ln}K_{A}=-\Delta H/RT+\Delta S/R$$ (4)

The calculated results corresponding to fitting of Stern-Volmer equation at different temperatures are listed in Table 1. It is clear that the bindings of both NSC48693 and NSC290956 to HSA are exothermic processes accompanied by negative values of Δ*G* and positive values of Δ*S*. Especially, both negative values of Δ*G* at various temperatures imply the tendency of spontaneous binding of NSC48693 and NSC290956 to HSA. Moreover, the negative *ΔH* (-19.03 kJ mol^-1^) and positive *ΔS* (15.27 J mol^-1^ K^-1^) values show that electrostic attractions play a key role in the binding of NSC48693 to HSA and enthalpy driven is a characteristic sign during the binding interaction. Meanwhile, both positive values of *ΔH* (12.28 kJ mol^-1^) and *ΔS* (119.29 J mol^-1^ K^-1^) mean that hydrophobic interaction plays a vital role in the binding of NSC290956 to HSA. In addition, positive *ΔS* may also be a sign of electrostatic interaction [18], which means that electrostatic interactions also participate in the binding interaction between NSC290956 and HSA. Nevertheless, it is noteworthy to mention that the major contribution to Δ*G* arises from *TΔS* rather than *ΔH*, so the binding process is entropy driven.

**Table 1:** Thermodynamic parameters of binding of anticancer leads to HSA.

Compound	T (K)	ΔG (kJ mol^-1^)	ΔH (kJ mol^-1^)	ΔS (J mol^-1^ K^-1^)
NSC48693	293	-23.49	-19.03	15.27
	298	-23.58		
NSC290956	293	-22.67	12.28	119.29
	298	-23.26		

ITC is a gold standard for characterization of biomolecular recognition. It provides the information on number of binding sites, magnitude of binding affinity and two thermodynamic terms: Δ*H* and Δ*S* [33]. In this study, ITC assay was used to capture the binding heat released from HSA bound anticancer leads. Furthermore, the binding heat data was used to quantify the corresponding thermodynamic parameters. Since ITC assay is carried out often at higher concentrations of both proteins and ligands, ITC serves as a complement for fluorescence titration study, especially for weakly binding system [34].

**Fig 4:**
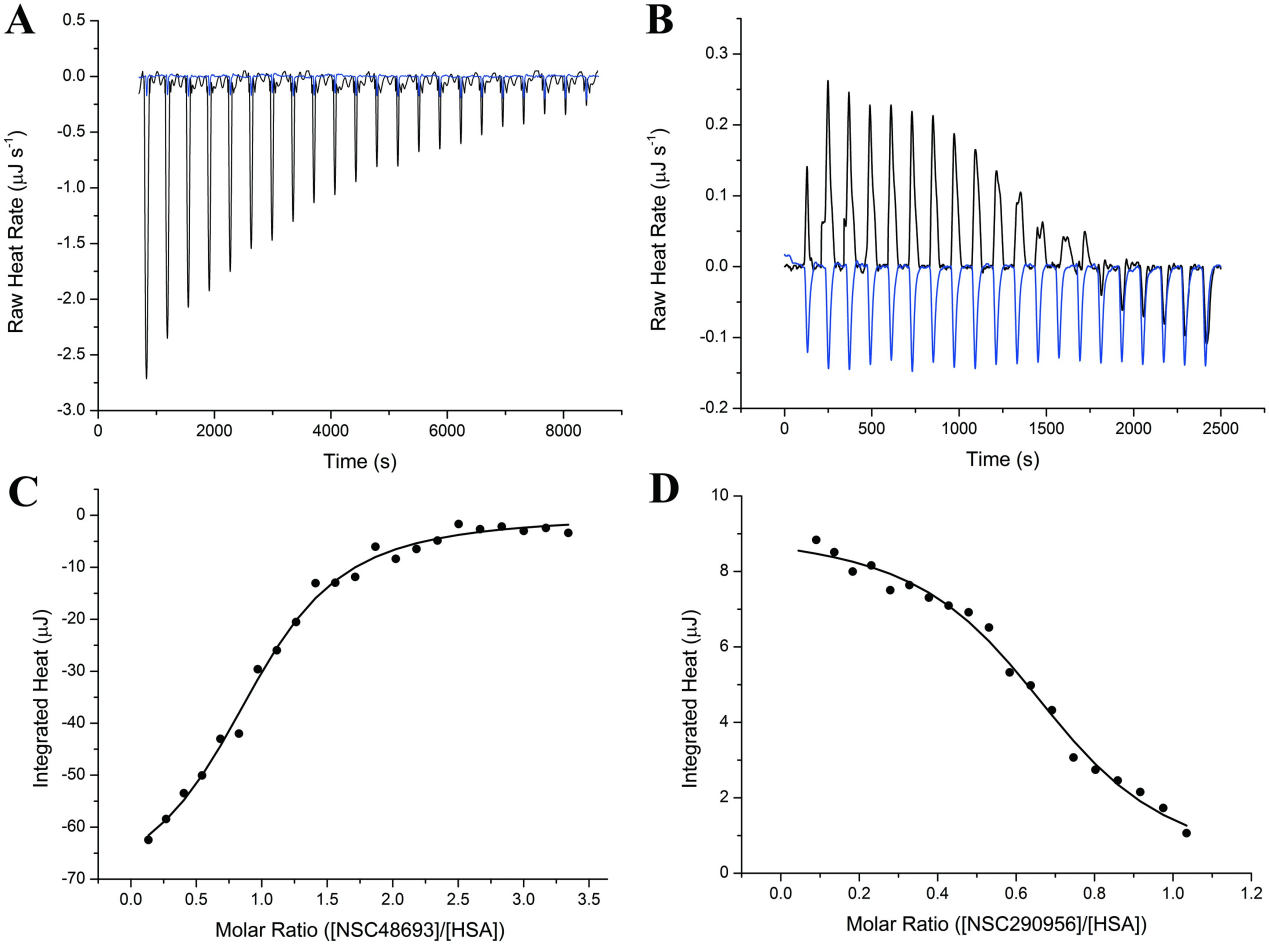
Binding thermodynamic measurements of NSC48693 (A, C) and NSC290956 (B, D) interacting with HSA measured by ITC. (A) and (B): raw heat released belonging to each injection (dilution spike of blank titration shown in dark blue lines). (C) and (D): integrated heat corresponding to each injection. The solid line shows the best fitting of integrated heat to a 1:1 isothermal binding model. Experiments were performed at 310 K as described in Materials and Methods.

Experimentally, the ITC assays measure the observable raw heat release by progressively titrating NSC48693 and NSC290956 into HSA solution (Fig 4A and 4B). The integrated raw heat can be well fitted to one-site independent isothermal binding model as displayed in Fig 4C and 4D. The binding constant (*K*_*A*_) values, 7.29 ± 2.68 × 10^4^ M^-1^ for NSC48693 and 2.65 ± 0.63 × 10^4^ M^-1^ for NSC290956, are comparable to the values obtained by nonlinear regression fitting equation. The obtained Δ*H* (-23.35 ± 2.53 kJ mol^-1^ for NSC48693 and 12.07 ± 0.45 kJ mol^-1^ for NSC290956) and Δ*S* (17.81 J mol^-1^ K^-1^ for NSC48693 and 140 J mol^-1^ K^-1^ for NSC290956) are also within the same magnitude when compared with temperature-dependent fluorescence quenching analysis listed in Table 1. The apparent Δ*H* and Δ*S* values determined by both fluorescence quenching and ITC method have the similar values. Both Δ*G* values (-28.86 kJ mol^-1^ and -32.19 kJ mol^-1^ for NSC48693 and NSC290956 at 310 K, respectively) are less than those calculated data based on the temperature-dependent fluorescence quenching titration assays at 293 K and 298 K as listed in Table 1. However, the Δ*G* value obtained from ITC assay is in good accordance with the tendency, namely the Δ*G* value is reduced with the temperature increase in the fluorescence quenching titration experiment. The stoichiometry numbers (0.94 ± 0.06 for NSC48693 and 0.70 ± 0.02 for NSC290956), correlated to the Hill coefficient *h*, are also in good accordance with the values obtained by Hill equation (*n* = 0.91 for NSC48693 and *n* = 0.75 for NSC290956). In NSC48693-HSA binding system, (i) Δ*H* has a negative value, and (ii) the absolute value of Δ*H* is larger than the multiplication of temperature (310 K) and Δ*S*. The two factors collectively lead to the enthalpy driven binding of NSC48693 to HSA. In other words, the electrostic interaction between NSC48693 and HSA mainly contributes to the ligand-protein complex formation. On the contrary, Δ*H* and Δ*S* are all positive values, suggesting that hydrophobic interaction dominates the binding between NSC290956 and HSA. Additionally, the value of TΔ*S* is larger than that of Δ*H*, which ultimately leads to a negative Δ*G* during the binding of NSC290956 to HSA. Thus, the binding is a entropy driven process. All these results are reasonably in support of the fluorescence quenching analysis.

### 4. Fluorescence resonance energy transfer

**Fig 5:**
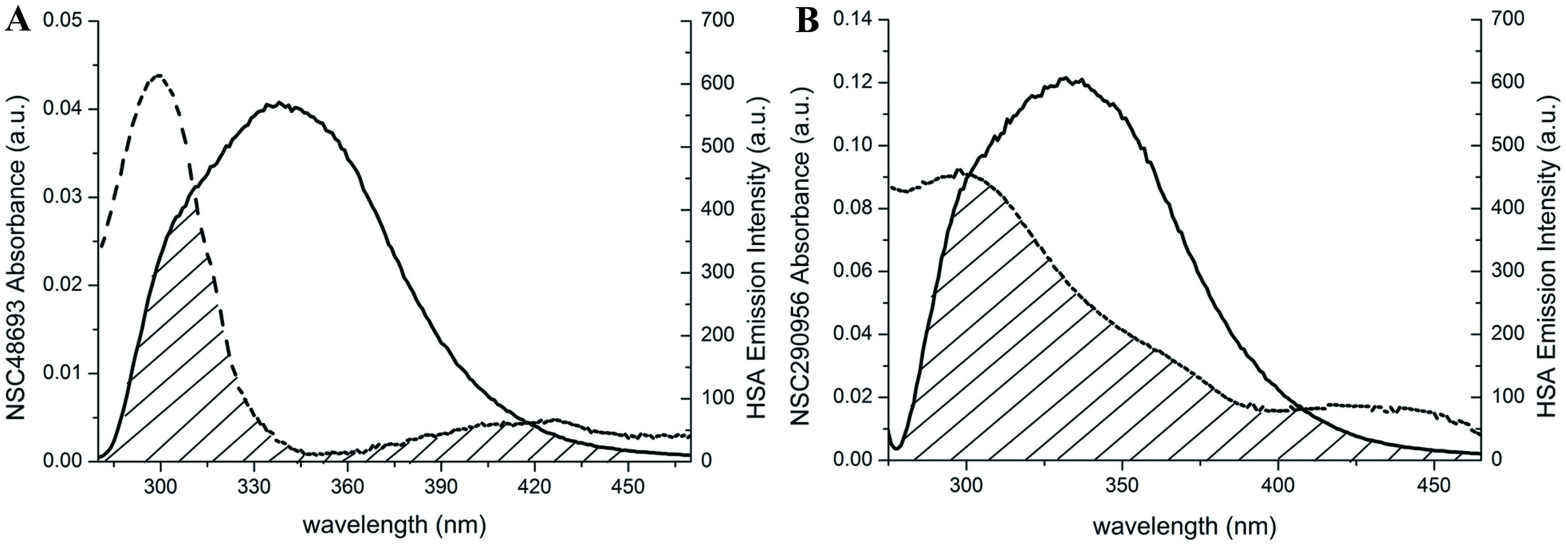
Overlapping of the fluorescence emission spectrum of HSA with UV-vis absorption spectra of NSC48693 (A) and NSC290956 (B) at corresponding wavelength. Overlapping area is marked with shadow lines.

Fluorescence resonance energy transfer (FRET) occurs when the UV absorption spectrum of small molecule (acceptor) overlaps with the fluorescence emission spectrum of fluoreophore (donor). The fluorescence emission peak of HSA mostly locates in the range of the UV-vis absorption spectra of the two anticancer leads (Fig 5A and 5B). This indicates the possibility of FRET occuring between HSA and anticancer leads. According to Forster’s non-radiative energy transfer theory, the distance *r* between donor W214 of HSA and the acceptor of small molecule is determined by the relationship below:

$$E=1-\frac{F}{F_{0}}=\frac{R_{0}{}^{6}}{R_{0}{}^{6}+r^{6}}$$ (5)

where *E* is the energy transfer efficiency; *F* and *F*_*0*_ are the fluorescence intensity in the presence and absence of quencher. The critical distance *R*_*0*_, the 50% energy transfer efficiency distance between the donor and acceptor, can be calculated with the equation:

$$R_{0}{}^{6}=8.8\times 10^{-25}k^{2}N^{4}\Phi J$$ (6)

where *k*^2^ is the spatial orientation factor related to the geometry of the donor and acceptor of dipoles; *N* is the refractive index of the medium; Φ is the fluorescence quantum yield of the donor; and *J* shows the degree of spectral overlap between the total fluorescence emission spectrum of the donor and the UV-vis absorption spectrum of acceptor. In this case here, *k*^2^ = 2/3 is for random orientation as in fluid solution, *N* = 1.36, Φ = 0.15, and *J* is calculated as:

$$J=\frac{\sum F\left(\lambda \right)\varepsilon \left(\lambda \right)\lambda^{4}\Delta \lambda }{\sum F\left(\lambda \right)\Delta \lambda }$$ (7)

where *F*(*λ*) is the fluorescence intensity of the fluorescence donor of wavelength *λ*; and ε(*λ*) is the molar absorption coefficient calculated by Lambert-Beer relationship ε(*λ*) = *A*(*λ*)/*cl* at wavelength *λ*. The integration step was chosen to be the scanning data interval of 1 nm.

The obtained donor-acceptor distances are less than 10 nm, the maximum distance of FRET happening. In particularly, the distance between HSA and NSC48693 is 3.65 nm that is far less than the diameter of HSA (8 nm × 8 nm × 3 nm) [35], suggesting that the energy transfer between NSC48693 and HSA occurs with high possibility. However, the distance (7.81 nm) between HSA and NSC290956 is slightly less than the diameter of HSA, which means that the energy transfer between NSC290956 and HSA occurs with little possiblity.

### 5. Effect of anticancer leads on HSA conformation

**Fig 6:**
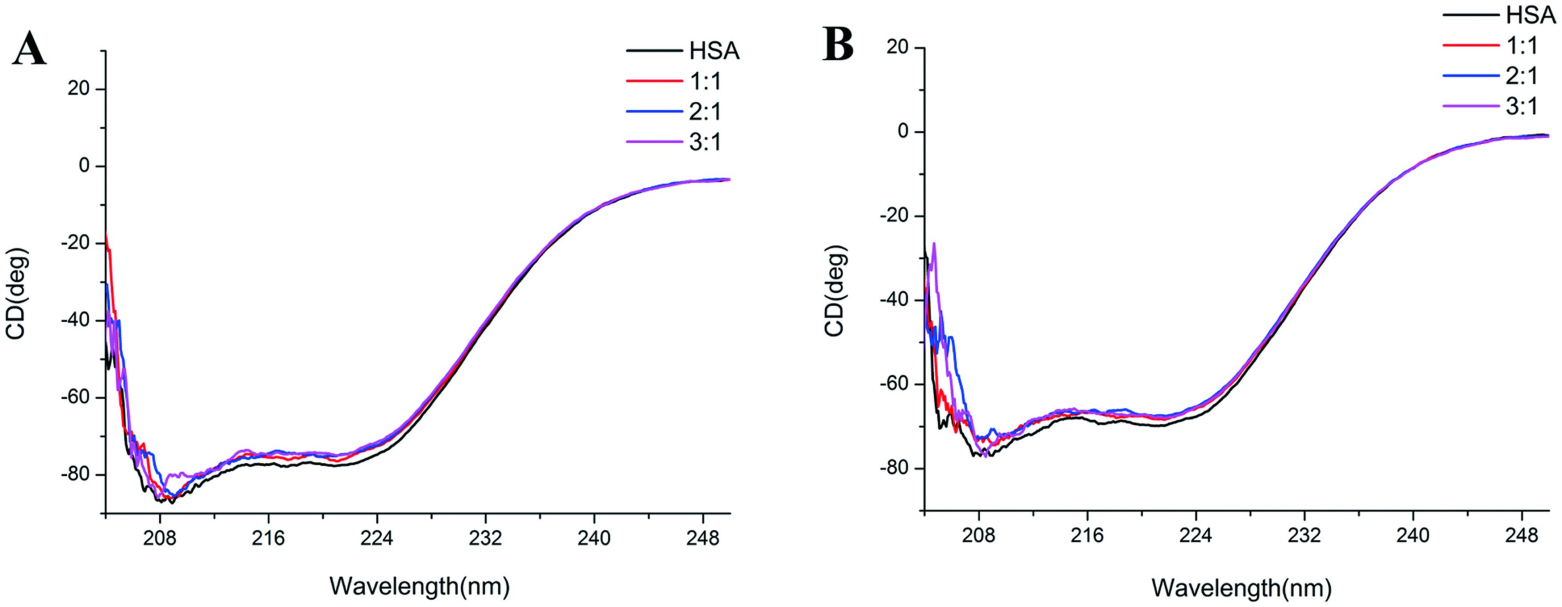
Effect of anticancer leads-bound on HSA conformation. Far-UV CD spectra of NSC48693 (A) and NSC290956 (B) binding to HSA. [HSA] = 2 μM. Concentration ratio of [small molecule]: [HSA] is shown in colored lines.

CD is a sensitively spectroscopic method extensivley used for quantifying protein structure change [36]. Far-UV CD (190–250 nm) typically uncovers the protein secondary structure change, since the two characteristic bands located at 208 nm and 222 nm represent the characteristic of α-helical protein [37]. Herein, the conformation changes accompanied by anticancer leads binding have been examined by using far-UV CD in the range of 200–250 nm. When these two anticancer leads were added into HSA solution at various concentration ratio (1:1, 2:1, 3:1), there is a slight decrease of α-helix for HSA conformation (Fig 6A and 6B). Possibly even more important is the interactions of both NSC48693 and NSC290956 with HSA inducing secondary structure unwinding.

### 6. Kinetic behavior of anticancer leads binding HSA

**Fig 7:**
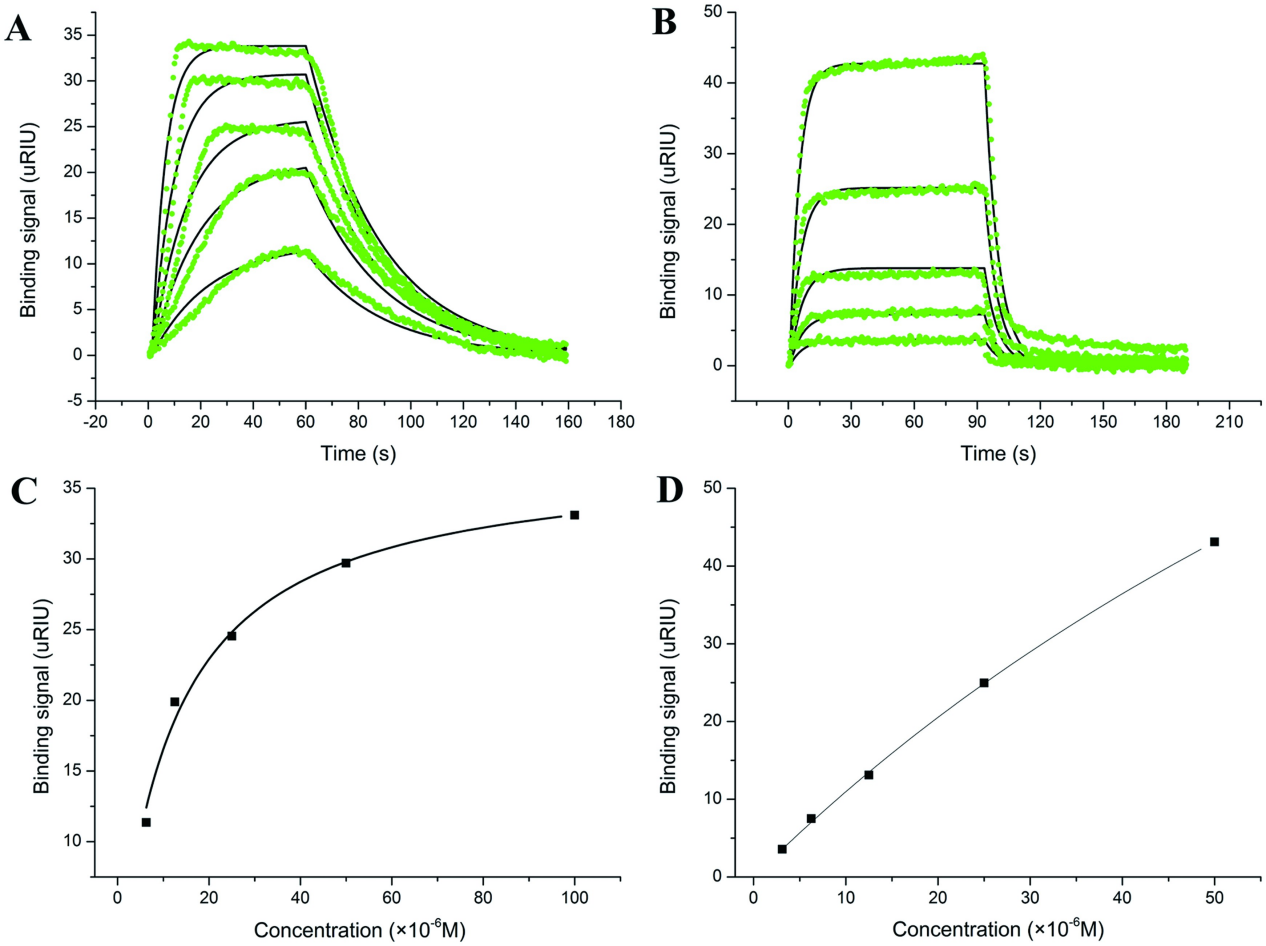
Kinetic characterization of anticancer leads binding with HSA. Kinetic signal response determined by SPR is recorded as the difference between the index of refraction of the sample and the index of refraction with buffer. The micro refractive index units (μRIU) is represented by green lines. The best fitting line of 1:1 binding model is shown for NSC48693 (A) and NSC290956 (B) with black solid line. The saturated signals for NSC48693 (C) and NSC290956 (D) are fitted with one step equilibrium binding model.

Lable-free optical biosensor based on SPR has been extensively used to study kinetic behavior of anticancer leads binding target proteins [38, 39]. In this case, SPR method was used to extract both the kinetic and thermodynamic parameters during the binding interactions of anticancer leads with HSA. The binding responses, when anticancer leads were injected to immobilized HSA, are reported in Fig 7A and 7B. In both cases, a significant concentration-dependent manner is observed. The *K*_*D*_ values obtained by steady-state phase data (1.38 × 10^−5^ M for NSC48693 and 1.16 × 10^−4^ M for NSC290956) and by dividing kinetic parameters (1.23 × 10^−5^ M for NSC48693 and 1.35 × 10^−4^ M for NSC290956) are all in reasonable accordance with the fluorescence titration results. As displayed from the square wave shape of sensorgram, NSC48693 exhibits fast-on and slow-off kinetic behavior. On the contrary, interaction of NSC290956 with HSA reaches steady-state within a few seconds from the injection and rapidly dissociates from binding site, exhibiting fast-on and fast-off kinetic behavior. Both association and dissociation rate constants were obtained by fitting the square wave shape of the sensorgrams to a 1:1 single step kinetic model as shown in Fig 7C and 7D. All the kinetic parameters including kinetic rate constant and thermodynamic *K*_*D*_ for the binding interaction are given by the data summarized in Table 2. It should be noted that when performing SPR, the protein was covalently linked to the optical sensor chip, otherwise there would not be any sensible signal produced. The protein immobilized on the surface of the sensor chip causes the reduction of protein rotation freedom of degree, which further results in the decrease in accesible binding sites when compared to the free protein in the solution-phase [40]. This random orientation of immobilizaed molecules causes systematic underestimation of the association constants of small molecules binding to proteins. However, given the available number of linkage groups on the protein surface, the measured results still have reasonable accuracy for drug-protein binding study. Thus, the SPR assay revealed that the affinity of anticancer leads binding to HSA is in reasonable agreement with the results obtained from fluorescence titration and ITC assays. It is clearly shown that NSC290956 exhibits slower association rate concomitant with a faster dissociation rate, thus could be more easily released to target organ.

**Table 2:** Kinetic parameters by fitting SPR data to 1:1 model.

Compound	K_D_ (M)	K_a_ (M^-1^ s^-1^)	K_d_ (s^-1^)	Steady State K_D_ (M)
NSC48693	1.38×10^−5^	2.41×10^3^	3.33×10^−2^	1.23×10^−5^
NSC290956	1.16×10^−4^	1.32×10^3^	1.53×10^−1^	1.35×10^−4^

### 7. Confirmation of anticancer leads-HSA complexes by molecular modeling

**Fig 8:**
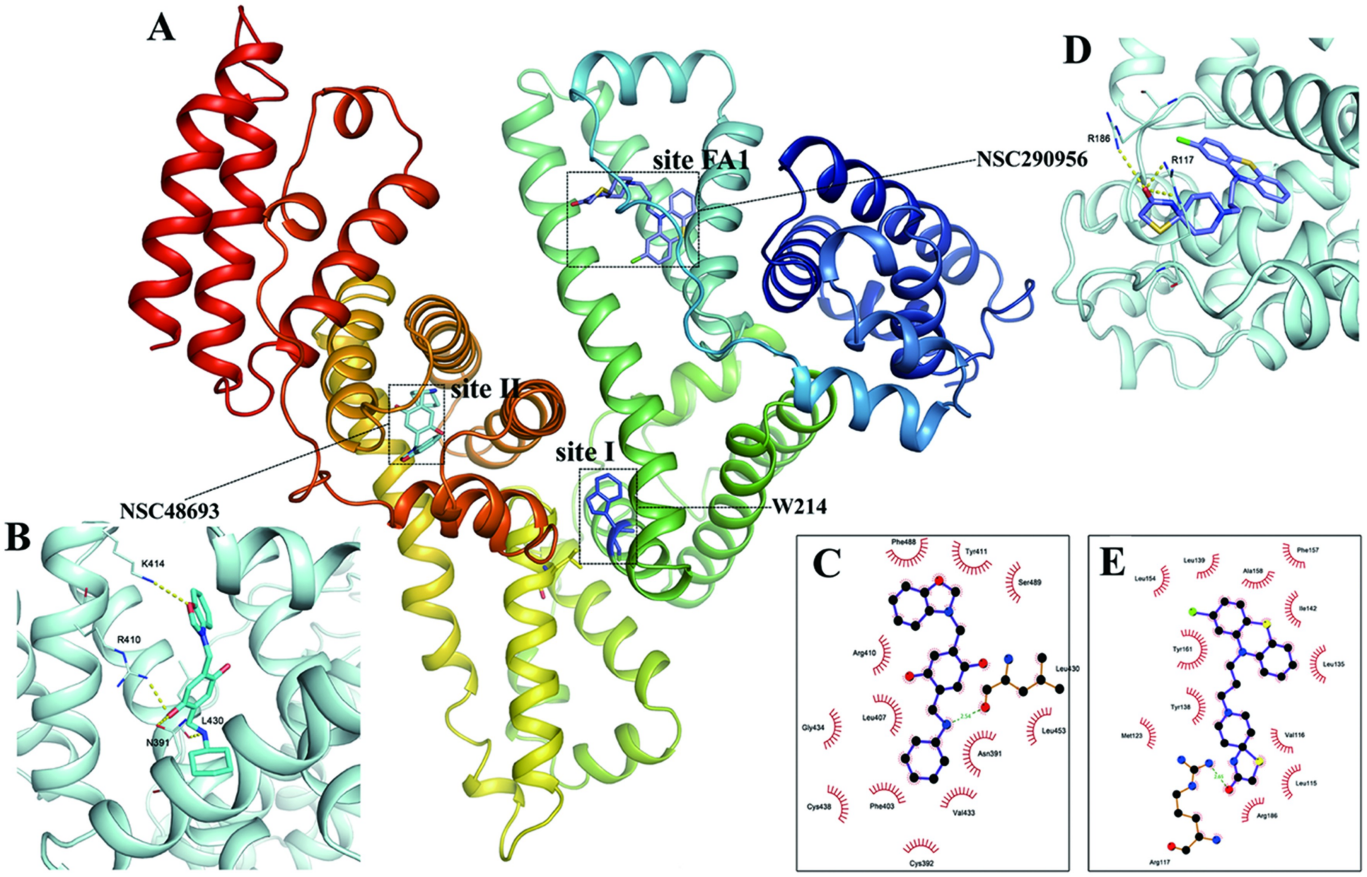
Molecular modeling of NSC48693 and NSC290956 to HSA at the respective binding site. Docking molecules are emphasized with dashed frame, and the detailed binding site structures and surrounding hydrophobic residues are linked by dashed blank lines. In site structure view, possible hydrogen bonds are shown in dashed yellow lines. A: HSA structure and elucidation of drug binding site; B and C: detailed structure and surrounding hydrophobic residues of NSC48693 binding site; D and E: detailed structure and surrounding hydrophobic residues of NSC48693 binding site.

Accumulating biochemical evidence indicates the validity of the binding between both leads and HSA in experimental ways. To further understand both leads binding with HSA, experimental observations were interpreted on the basis of molecular docking. In the process of docking, 500 conformations of each ligand were generated and selected. Cluster analysis revealed the most probable conformation cluster (Fig 8A) with the minimum energy of -8.81 kcal mol^-1^ for NSC48693 and -12.59 kcal mol^-1^ for NSC290956, respectively. As seen in Fig 8B, NSC48693 forms possible hydrogen bonds with residue N391, R410, K414 and L430 located at the entrance of site II. This binding model exhibits large enthalpy contribution to the complex formation. This observation is in agreement with the conclusions described above using ITC and fluorescence quenching measurements. Moreover, since NSC48693 is soluble in water, this hydrophilic property leads to a small entropy change. The binding site comprises of several nonpolar amino acid, such as Asn391, Phe403, Leu407, Arg410, Tyr411, Val433, Leu453 and Phe488 (Fig 8C). They contibute to a positive entropy change value, which is in accordance with experiment results. The distance of NSC48693 to W214 is 3.426 nm, in accordance with the calculated FRET distance, which further confirms that the binding site is located at drug site II as shown in the docking conformation. In contrast, NSC290956 forms possible hydrogen bond with R117 and R186 at fatty acid 1 (FA1) binding site (Fig 8D), and is surrounded with numbers of nonpolar amino acid residues, such as Leu115, Val16, Leu135, Tyr138, Leu139, Ile142, Ala158, Tyr161, Arg186 and Ile (Fig 8E). Given the hydrophobic property of NSC290956, the binding is essentially entropy driven with the feature of hydrophobic force domination. This is in accordance with experimental results. Accordingly, these findings not only provide an optimal structural basis to explain HSA fluorescence quenching in the presence of both NSC48693 and NSC290956, but also supplement the formation of intermolecular H-bonds that cannot be probed directly by the experimental method.

## Discussion

Information on the affinity of drug candidates to plasma proteins is essential in the drug discovery and development process. HSA is known to bind different classes of small molecules at various sites and therefore is considered as a potential molecular cargo for clinical or biophysical purposes. Binding affinity to proteins can also be determined by various approaches, depending on a change in a physicochemical property of either the ligand or the protein due to the binding. Spectroscopic techniques including fluorescence, UV-vis absorption and CD are frequently used. Other widely implemented methods, mainly for investigation of binding energetics and binding kinetics, are ITC and SPR methods.

To understand the molecular and physiological processes of two promising anticancer leads interacting with HSA, we performed the binding experiments of hydrophilic NSC48693 and hydrophobic NSC290956 binding HSA in physiological conditions. It is well established that the presence of hydrophobic moieties as well as hydrophilic negatively charged groups are the basic structural requirements for ligand binding to HSA. The differences in behaviors are direct consequences of the interaction forces. In this case, NSC48693 is found to be tightly binding to HSA subdomain III A, which exhibits large enthalpy change via the formation of extensive hydrogen bonds with surronding amino acids. However, NSC290956 possiblely binds to FA1 site at subdomain I B on HSA, which is driven by entropy forces in the loop-helix. Based on the molecular chemical properties, hydrophilic drugs prefer to establish extensive hydrogen bonds with HSA, driving the compounds into the hydrophilic pocket to form tightly bound complex. It is clear that the hydrophilic NSC48693 tends to have larger dissociation rate constants since the disruption of hydrogen bonds requires considerable energy. In contrast, the binding of hydrophobic NSC290956 with HSA is promoted by hydrophobic forces. Therefore, the formation of drug-protein complex is an entropy driven process. The binding site is localized in a hydrophobic pocket to achieve maximum entropy benefits, leading to modest association strength and large dissociation rate constants.

The function of HSA as drug carrier may facilitate drug access to the action site and be related to drug safety issues. Application of HSA on drug delivery may therefore be important in anticancer therapy, which has been taken into account in estimating therapeutic outcomes, particularly for highly bound compounds (defined as < 5% free drug compounds in blood). The free to bound drug rates can be estimated by using the formula K_d_ / ([P]_f_ + K_d_) through approximating the free protein concentration as [P]_f_ = [P]_T_ − [L]_T_ [41]. Since the typical plasma protein concentration is about 0.5 M, the order of 10^4^ in association constants of NSC48693 and NSC290956 indicates that over 98% of compounds are bound to plasma proteins. The binding affinity of NSC48693 to HSA is almost 3~8 fold larger than that of NSC290956 binding HSA as measured with different experimental methods. This affinity difference may be interpreted by the smaller molecular weight of NSC48693, which can be buried deeper to form more hydrogen bonds [42]. The binding affinity exhibits the free energy change of the drug-HSA association and is thermodynamically correlated with the equilibrium association/dissociation constant. Therefore, the affinity determines the steady state concentration of unbound or exposed drug in the plasma, maintaining a concentration gradient between the intestinal lumen and plasma, which is considered as the only fraction exerting the bioactivity. Thus, the high affinity of NSC48693 binding to HSA makes it easier to be absorbed by oral administration. For NSC290956, since drug-HSA binding results in increased solubility for lipophilic drugs, the absorption is also increased when drug enters plasma. Moreover, both hepatic uptake and glomerular filtration are directly proportional to the free drug fraction presented in the plasma while drugs are modulated by the affinity difference of the plasma and tissue proteins [43]. If a drug is highly bound to HSA and has a high extraction raio, the binding promotes metabolism by transporting the drug to liver, resulting in the decrease of free drug fraction. On the other hand, if a drug has a low liver extraction ratio, metabolism will be decreased by high HSA binding. The situation is the same for kidney elimination, since drugs bound to HSA cannot undergo glomerular filtration, the half-life of drugs in plasma is increased. Similarly, high renal extraction ratio and high HSA binding promote elimination by kidney. Herein, stronger binding of NSC48693 potentially increases not only the drug’s plasma half-life but also the risk of high metabolism and elimination ratio. Moreover, drugs that exhibit very tight bound to HSA usually have low distribution volume because the strong association confines drugs to the vascular space [44].

Apart from the binding stability of drug to HSA, the association and dissociation rate constants also deeply affect the pharmacodynamic efficacy on several aspects. The association rate determines how fast the drug-HSA complex form, while the average bound time is determined by the reciprocal of the off-rate constant. Therefore a smaller value of the off-rate constant will achieve longer residence time for a similar affinity [44]. Herein, although the association rates are similar, the dissociation rate for NSC290956 is much faster (almost one order magnitude) than that for NSC4893. Accordingly, the smaller dissociation rate of NSC48693 results in a longer average time interval of binding HSA, which is beneficial for drug’s plasma half-life [45]. To achieve high selectivity to target tissues, it is necessary to involve the enhanced permeability and retention (EPR) effect, which facilitates drug accessing to the action site, reducing the side effects and enhancing pharmacodynamic efficacy. It has been clearly demostrated that EPR effect-driven drug delivery does not occur in normal tissues, although EPR gives molecular size-dependent effect [46]. Due to rapid diffusion and renal clearance, EPR effect does not apply to low-molecular weight drugs. Thus, it can be concluded that plasma protein binding becomes the only possible way to increase selectivity [47]. HSA has a molecular weight of about 66 KDa that is above the threshold of renal clearance, which contributes to the selective accumulation effect in tumor tissues. The accumulation degree is far more than that observed in normal tissues according to EPR effect. As accumulation of HSA by EPR is a progressive phenomenon, desired concentration of drugs can only be realized by consistence dosage. Under this circumstances, a fast release rate from HSA produces a large fraction of exposed drug. This leads to little drug accumulation in tumor tissues, which ultimately results in a poor therapeutic outcome and an undesired toxicity. Rather, a slow release rate often results in an insufficient active drug concentration at action site that demands usually a high concentration dosage [6]. As far as NSC48693 and NSC290956 are concerned, the dissociation rate is medium, i.e. the dissociation rate values locate in a reasonable range [48]. We therefore draw a conclusion that NSC290956 can be more easily released from HSA to tumor tissues under the condition of consistence dosage administration due to the faster dissociation rate.

## Conclusions

In summary, the specific molecular structures and chemical properties of these two anticancer leads NSC48693 and NSC290956 lead to distinct molecular and physiological binding process as well as different equilibrium and kinetic parameters. These parameters influence the pharmacodynamic efficacy and pharmacokinetic behavior, which will give further impacts on drug efficacy *in vivo*. For NSC48693, the pharmacodynamic efficacy is less than that of NSC290956, while its pharmacokinetic behavior is better than that of NSC290956 if consistence drug dosage administration is not under consideration. Thus, several valuable information can be inferred from our current results for the next structure modification: (i) the dissociation rate of NSC48693 should be increased for achieving optimal drug concentration at the action site to improve its pharmacodynamics; (ii) the affinity of NSC290956 binding HSA should be increased to provide prolonging plasma half-life that leads to better pharmacokinetics. Taken together, our work uncovers a sufficiently accurate basis for offering a physical insight into the relation between drug chemical structure and pharmacodynamics/parmacokinetics.

## Supporting information

S1 Fig Chemical structure of NSC48693 (A) and NSC290956 (B). (DOCX)

S2 Fig Excitation and emission spectra of NSC48693 (A) and NSC290956 (B). (DOCX)

S3 Fig Steady-state fluorescence spectra of NSC48693 (A) and comparative plots of synchronous fluorescence intensity (Δλ = 60 nm) with (green dot) and without (blue dot) HSA (B). (DOCX)
