# Correction: Uncovering the molecular and physiological processes of anticancer leads binding human serum albumin: A physical insight into drug efficacy

**DOI:** 10.1371/journal.pone.0178660

**Published:** 2017-05-23

**Authors:** The PLOS ONE Staff

In the Author Contributions section, Jin Wang (JW) should be listed as one of the persons responsible for writing—reviewing & editing.

The following information is missing from the Funding Disclosure: This study was supported by National Science Foundation (grant no. PHY-76066).

The publisher apologizes for these errors.
