# Supplementary figures and images for: Uncovering the molecular and physiological processes of anticancer leads binding human serum albumin: A physical insight into drug efficacy
Source: PLoS One. 2017 Apr 20;12(4):e0176208. doi: 10.1371/journal.pone.0176208 (PMC5398698; doi:10.1371/journal.pone.0176208)

**
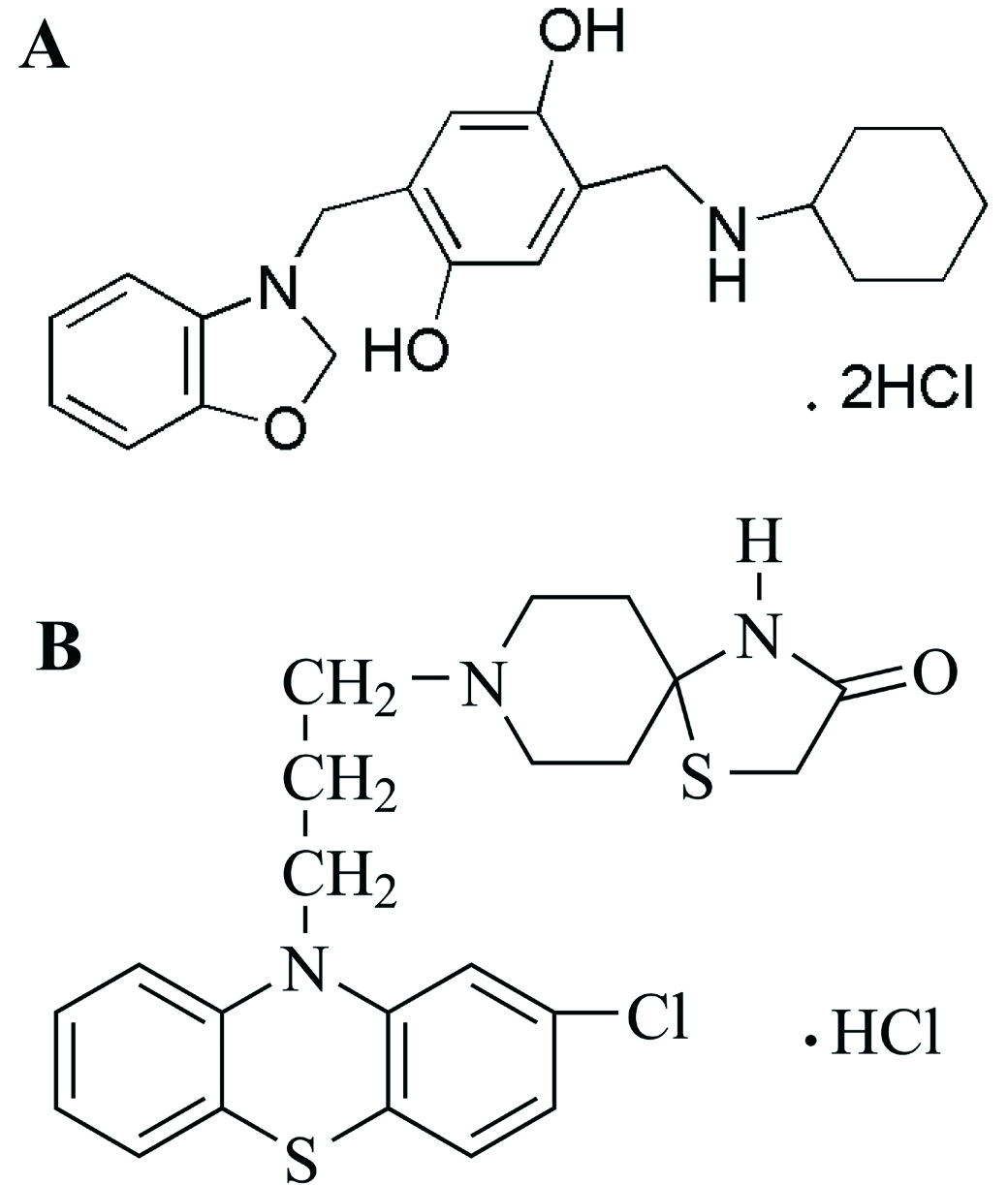
**

**S1 Fig**. **Chemical structure of NSC48693 (A) and NSC290956 (B)**.

Supplement: S1 Fig — (DOCX) [file pone.0176208.s001.docx]
