# Supplementary material for: Uncovering the molecular and physiological processes of anticancer leads binding human serum albumin: A physical insight into drug efficacy
Source: PLoS One. 2017 Apr 20;12(4):e0176208. doi: 10.1371/journal.pone.0176208 (PMC5398698; doi:10.1371/journal.pone.0176208)
Supplement: S2 Fig — (DOCX) [file pone.0176208.s002.docx]

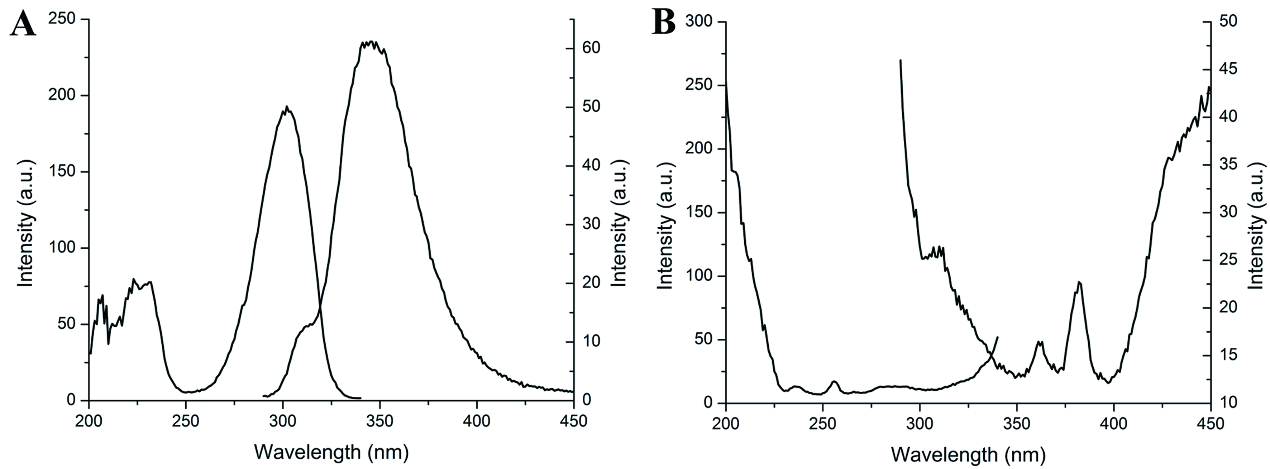


**S2 Fig**. **Excitation and emission spectra of NSC48693 (A) and NSC290956 (B)**. Experiments were performed at 300 K in 0.01 M PBS buffer (pH 7.4). The respective concentration of NSC290956 and NSC48693 was 100 μM. Excitation and emission slits were all kept at 5 nm. The excitation and emission pathlength was 1 cm and 0.2 cm, respectively. The excitation and emission wavelength was 280 nm and 350 nm, respectively.
