# Supplementary material for: Uncovering the molecular and physiological processes of anticancer leads binding human serum albumin: A physical insight into drug efficacy
Source: PLoS One. 2017 Apr 20;12(4):e0176208. doi: 10.1371/journal.pone.0176208 (PMC5398698; doi:10.1371/journal.pone.0176208)
Supplement: S3 Fig — (DOCX) [file pone.0176208.s003.docx]

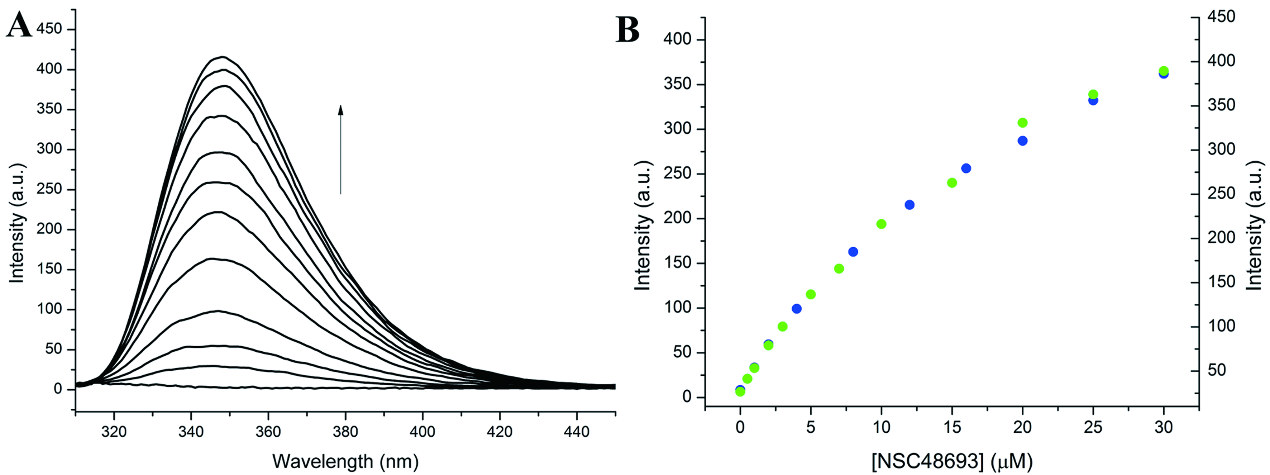


**S3 Fig.** **Steady-state fluorescence spectra of NSC48693 (A) and comparative plots of synchronous fluorescence intensity (Δλ = 60 nm) with (green dot) and without (blue dot) HSA (B)**. Fluorescence spectra was recorded by gradually adding NSC48693 into 0.01M PBS buffer (pH 7.4). Excitation wavelength was set to 295 nm with excitation and emission slit all kept at 5 nm. The axis of comparative plots was adjusted to emphasize the similarity of these two data plots.
